# Advertising Physicians

**Published:** 1880-06

**Authors:** 


ADVERTISING PHYSICIANS—We have never read the *code of ethics* of the American Medical Association through, and cannot consequently say, *how far* a Physician can go, in inviting attention to himself, and the honors and appointments he has received to do this, that or the other matter, for one or another society, as its representative, etc., or in making known *his* professional achievements and other exploits through the secular papers, without rendering himself amenable to its laws; but measured by the rules of *common decency*, one sees in the over-weening desire of certain members of the profession to attract attention to themselves, by supplying editors of secular newspapers with items of information in regard to them, sometimes doubtless, the fruit of a fawning sycophancy, that is violative, not only of professional dignity but so reprehensible as to evoke feelings of utter contempt for the authors of this kind of trickery, from all honorable minds. Such conduct is reprehensible in any calling, but particularly so in our profession, where culture and merit, and not *clandestine advertising* through the local columns of a secular newspaper, should be the measure of one's success in attracting public patronage and influence to him in his community or neighborhood.

We are forcibly reminded of stealing the livery of Heaven to serve the devil in; when paragraphs of the character we are animadverting upon, come under our observation. It were far better there were no medical societies, nor *codes of ethics* at all, than that *such members* should be allowed to *disgrace the one and treacherously defy the other.*

